# Effects of EPA+DHA and Corn Oil Supplementation on PUFA Concentrations across Plasma Lipid Pools and on Downstream Oxylipins: Exploratory Results from a Randomized Controlled Trial in Healthy Humans

**DOI:** 10.1016/j.tjnut.2025.101274

**Published:** 2025-12-27

**Authors:** Neha Balakrishnan, Saame Raza Shaikh, Caroline E Childs, Elizabeth A Miles, Paul S Noakes, Carolina Paras-Chavez, Michael Armstrong, Nicole Reisdorph, Philip C Calder, Helena L Fisk

**Affiliations:** 1 Department of Nutrition, Gillings School of Global Public Health and School of Medicine, University of North Carolina at Chapel Hill, Chapel Hill, NC, United States; 2 School of Human Development and Health, Faculty of Medicine, University of Southampton, Southampton, United Kingdom; 3 School of Medicine, University of Notre Dame Australia, Fremantle, Australia; 4 Department of Pharmaceutical Sciences, University of Colorado-Anschutz Medical Campus, Aurora, CO, United States; 5 National Institute for Health and Care Research Southampton Biomedical Research Centre, University Hospital Southampton NHS Foundation Trust and University of Southampton, Southampton, United Kingdom

**Keywords:** docosahexaenoic acid, eicosapentaenoic acid, oxylipins, fatty acids, inflammation

## Abstract

**Background:**

Eicosapentaenoic acid (EPA) and docosahexaenoic acid (DHA) may improve inflammatory conditions. We previously demonstrated that supplementation with EPA+DHA in adults elevates anti-inflammatory oxylipins in human plasma and adipose tissue. However, the localization of EPA/DHA in plasma lipid pools [phosphatidylcholines (PC), triglycerides (TAG), cholesteryl esters (CE), and nonesterified fatty acids (NEFA)] and how this relates to the downstream oxylipin levels remains unknown.

**Objectives:**

This study aimed to identify the incorporation of supplemental EPA+DHA into plasma PC, TAG, CE, NEFA, and the impact on downstream oxylipins.

**Methods:**

We conducted an exploratory analysis with available samples (*n* = 21, 20 female, 1 male, age 35–49 y) from a previous double-blind, placebo-controlled trial of participants randomly assigned to consume either 3 g of EPA+DHA concentrate (1.1 g EPA + 0.8 g DHA) or corn oil (CO) [1.65 g linoleic acid (LA) + 0.81 g oleic acid] daily for 12 wk. Plasma was analyzed using gas chromatography and mass spectrometry to quantify fatty acids and oxylipins, respectively.

**Results:**

EPA+DHA supplementation increased EPA levels across PC, CE, NEFA, and TAG pools, and increased DHA levels in PC, CE, and TAG pools. Conversely, supplementation decreased LA levels in PC, CE, and NEFA pools, and decreased arachidonic acid (AA) levels in PC and NEFA pools. EPA+DHA supplementation also led to significant shifts in oxylipin concentrations compared with baseline, with predominant increases in anti-inflammatory and decreases in proinflammatory oxylipins. CO supplementation decreased TAG AA levels and modified concentrations of several AA-derived oxylipins. Levels of EPA+DHA and derived oxylipins were significantly higher across lipid pools following supplementation with EPA+DHA compared with CO.

**Conclusions:**

These findings offer insights into supplemental EPA+DHA localization to different circulating lipid pools, which have implications for understanding how to mitigate systemic inflammation. Furthermore, studies are needed to evaluate relationships between the changes in polyunsaturated fatty acids, oxylipins, and markers of inflammation.

The study was registered at www.isrctn.com as ISRCTN96712688.

## Introduction

Chronic low-grade inflammation is a key contributor to the development and progression of a wide range of diseases [1]. In the United States, the prevalence of chronic inflammatory conditions has risen steeply, with nearly 60% of Americans having ≥1 condition involving inflammation, and 42% having >1 such condition [2]. This increase coincides with adopting the modern Western diet, which differs from the nutritional patterns consistent throughout human evolution [3]. Characterized by processed foods, high saturated fat, and a skewed omega (ω)-6 to ω-3 fatty acid ratio, this shift has occurred within a few generations [3,4]. However, high levels of ω-6 PUFAs may not be the cause of chronic low-grade inflammation, as many studies have shown the benefits of these PUFAs for health compared with the intake of saturated fatty acids [5]. For instance, moderate intake of linoleic acid (LA) lowers total cholesterol by promoting hepatic LDL clearance through upregulating LDL receptor expression and upregulating bile acid synthesis [6]. Rather than a high intake of ω-6 PUFAs being the main concern, the low intake of ω-3 PUFAs may be a more significant factor in creating a persistent proinflammatory state [1,4].

The long-chain ω-3 PUFAs, EPA and DHA, have emerged as modulators of inflammation [5,7]. On the basis of a comprehensive umbrella meta-analysis, EPA+DHA supplementation significantly reduced circulating concentrations of key inflammatory markers, including C-reactive protein, TNF-α, and IL-6 in patients with type II diabetes [8]. Another prospective study found that EPA+DHA supplementation (3.2 g/d of EPA and 1.6 g/d of DHA for 3 mo) significantly reduced insulin resistance, circulating triglycerides (TAG), and inflammatory markers in women with obesity and healthy controls [5]. The supplementation also downregulated the expression of key inflammatory transcripts in CD4+ T-cells [5]. Some benefits, such as reduced insulin, persisted for a month after stopping supplementation, whereas TAG levels and inflammatory markers reverted to baseline [5]. At a molecular level, dietary supplementation with EPA+DHA modulates inflammation by targeting ω-6 PUFA levels and metabolism [6]. Displacement of ω-6 PUFAs by ω-3 PUFAs reduces the production of proinflammatory oxylipins derived from arachidonic acid (AA) [6]. EPA and DHA generate a complex range of oxylipins with diverse immunomodulatory effects. These changes, along with the ability of ω-3 PUFAs to modulate intracellular signaling, gene expression, and cytokine production, contribute to their anti-inflammatory effects [8,9].

Our previous research examined the impact of EPA and DHA supplementation on the lipidome of subcutaneous white adipose tissue (scWAT) [9]. Oxylipin profiling identified 111 fatty acid metabolites in scWAT, with 12 wk of 1.1 g EPA + 0.8 g DHA daily producing changes in these oxylipin profiles: there was a significant decrease in several AA-derived metabolites, including 20-COOH-AA, 14-15-dihydroxyeicosatrienoic acid, and leukotriene (LTE) 4, and an increase in several EPA- and DHA-derived metabolites [9]. These results led to the present study, which is an exploratory analysis aimed at examining the circulating lipidomic profile following EPA+DHA or corn oil (CO) supplementation. The primary outcome is the change in PUFA levels across plasma lipid pools, including PC, CE, NEFA, and TAG. These lipid pools were specifically chosen because each plays a vital role in lipid metabolism and inflammation. PCs are the primary target of phospholipase activity during inflammation, fueling the production of inflammatory metabolites [10]. CEs, particularly when oxidized, contribute to atherosclerosis by activating macrophages and promoting the release of cytokines [11]. NEFAs can directly activate inflammatory pathways and impair insulin signaling at high concentrations [12]. TAGs are the primary storage form of fatty acids, high circulating levels have also been linked to an increased risk of inflammatory diseases [13]. Although prior research has shown that ω-3 PUFA supplementation can replace and lower ω-6 PUFA levels in phospholipids, few studies have examined whether this replacement also occurs in distinct pools [14,15].

## Methods

### Participants

One hundred individuals aged 18–65 y were recruited into a double-blind placebo (comparator oil) controlled trial at the University of Southampton, United Kingdom between 2010 and 2015. Sixteen individuals withdrew, leaving 39 healthy-weight individuals and 45 individuals living with obesity to complete the study. After completing the study’s primary and secondary outcomes, plasma was available from 21 individuals, a mix of healthy-weight individuals and individuals living with obesity. The current study generated plasma PC, NEFA, CE, and TAG fatty acid profiles, as well as plasma oxylipin profiles, in the 21 individuals. All 21 participants had complete plasma samples at both baseline and week 12 and were included in all analyses. Due to the exploratory nature of this study, the sample size was determined by the availability of sufficient plasma for lipidomic analysis and was not based on a priori power calculation.

Individuals outside the defined age and anthropometric categories, diagnosed with metabolic disease (e.g. diabetes and cardiovascular disease) or chronic gastrointestinal problems (e.g. inflammatory bowel disease, celiac disease, and cancer), using prescribed medicine to control blood lipids, blood pressure, or inflammation, consuming more than one serving of oily fish per week (140 g cooked), taking fish oil or other oil supplements, who were pregnant or planning to become pregnant during the study period, or were participating in another clinical trial were not eligible to be included in the study. The flow of participants through the full study is depicted in Supplemental Figure 1, which also shows the participants involved in the current exploratory study. The full study was approved by the National Research Ethics Service South Central—Berkshire Research Ethics Committee (submission number 11/SC/0384) and was registered prospectively at www.isrctn.com (study ID: ISRCTN96712688).

### Study design

Fasted blood was collected at week 0 (baseline) and after 12 wk of intervention (week-12), during which participants were randomly assigned to consume either 3 g of EPAX6000 (Epax Norway AS), providing 1.1 g EPA + 0.8 g DHA or 3 g of CO (providing 1.65 g LA and 0.81 g oleic acid) per day. The EPA and DHA in the supplement used in this study were in TAG form. Ten individuals were in the EPA+DHA group, and 11 were in the CO group. Both oils were provided in 1 g soft gel capsules, and the full composition of each oil is previously described [[9], [16]]. Treatment group blinding, randomization, and supplement packaging were completed independently by the Research Pharmacy at University Hospital Southampton, Southampton, United Kingdom. Blinding was maintained until the completion of statistical analysis for all primary outcomes. Adverse events (AEs) were reported for the wider original study, which included repeated adipose tissue biopsy [9]. The majority of the AEs were not related to the study procedures or interventions, but several were related to bruising and bleeding at the adipose biopsy site, 1 was possibly related to cannulation for blood collection (arm pain at cannulation site), and 2 were possibly related to supplement consumption, which included headache and indigestion [9].

### Anthropometry

Height, weight, body composition, and waist and hip circumference measurements were made using a Seca stadiometer (Seca, Hamburg, Germany Seca), bioelectrical impedance apparatus (TANITA BC-418), and a tape measure, respectively, as previously described [16].

### Sample preparation

Approximately 5 mL of heparinized blood was collected and stored on ice. Plasma was prepared by centrifugation (1900 × *g*, 10 min, room temperature) and stored at –80°C until analysis.

### Fatty acid composition and oxylipin analyses

Approximately 500 μL plasma was used for fatty acid extraction with the following internal standards added: 15:0 PC, 21:0 NEFA, 17:0 CE, and 15:0 TAG (Sigma-Aldrich). Total lipids were extracted from plasma and separated into PC, NEFA, CE, and TAG using solid phase extraction (SPE) with aminopropyl silica columns (Agilent) as previously described with serial elution using chloroform, chloroform:methanol (60:40 v/v), chloroform:methanol:glacial acetic acid (100:2:2 v/v), and hexane [17]. Fatty acid methyl esters were prepared from each lipid fraction and were separated by gas chromatography on a BPX-70 fused silica capillary column (30 m × 0.2 mm × 0.25 μm; manufactured by SGE) in an HP6890 gas chromatograph fitted with a flame ionization detector. Run conditions were as described in detail elsewhere [16,17].

Approximately 100 μL of plasma was used for oxylipin extraction as previously described with the following internal standards added: 5(S)-hydroxyeicosatetraenoic acid (HETE)-d8, 8-iso-prostaglandin (PGF)2a-d4, 9(S)-hydroxyoctadecadienoic acid (HODE)-d4, LTB4-d4, LTD4-d5, LTE4-d5, PGE2-d4, PGF2a-d9, and RvD2-d5 (Cayman Chemical) [18]. In brief, proteins were precipitated using ice cold methanol and supernatants were reconstituted in water:methanol (90;10 v/v) before SPE separation. Oxylipins were separated using Strata-X 33um 30 mg/1 mL SPE columns (Phenomenex) pretreated with 10% MeOH, by serial elution using 10% MeOH, methyl formate, and MeOH, drying each eluant under nitrogen then reconstituting in ethanol for analysis [18]. Quantitation of oxylipins was performed using 2D reverse phase HPLC Agilent 1260 (Agilent Technologies), tandem mass spectrometry (LC/MS/MS) (Agilent 6490). HPLC pump 1 buffers: 0.1% formic acid in water (solvent A) and 9:1 v:v acetonitrile:water with 0.1% formic acid (solvent B); pump 2 buffers: 0.01% formic acid in water (solvent C) and 1:1 v:v acetonitrile:isopropanol (solvent D).

Reconstituted oxylipins were injected on to an Agilent SB-C18 2.1×5 mm 1.8 μm trapping column with the following instrument protocol: pump 1: 2 mL/min for 0.5 min with 97% solvent A: 3% solvent B, switching valve changing to the trapping column from pump 1 to pump 2 at 0.51 min. The flow was reversed and the trapped oxylipins were eluted onto an Agilent Eclipse Plus C-18 2.1 × 150 mm 1.8 um analytical column using the following gradient at a flow rate of 0.3 mL/min: hold at 75% solvent A:25% solvent D from 0 min to 0.5 min, then a linear gradient from 25% to75% D over 20 min followed by an increase from 75% to 100% D from 20 min to 21 min, then holding at 100% D for 2 min. During the analytical gradient pump 1 washed the injection loop with 100% B for 22.5 min at 0.2 mL/min [18].

Mass spectrometric analysis was performed on an Agilent 6490 triple quadrupole mass spectrometer in negative ionization mode using the following instrument protocol: drying gas: 250°C at a flow rate of 15 mL/min; sheath gas: 350°C at 12 mL/min; nebulizer pressure: 35 psi; capillary voltage: 3500 V; data acquisition: dynamic multiple reaction monitoring mode using collision energies obtained by analysis of authentic standards [18].

### Statistics

All statistical analyses were conducted using GraphPad Prism version 10 for macOS (GraphPad Software). Baseline differences between the 2 groups were evaluated with an unpaired *t*-test, paired *t*-tests were used to compare preintervention and postintervention values within each treatment group, and analysis of covariance (ANCOVA) was used to compare values between the EPA+DHA and CO groups at week 12, correcting for baseline value. Paired *t*-tests were used to compare pre- and post-intervention values within each treatment group, and ANCOVA was used to compare values between the EPA+DHA and CO groups at week 12, correcting for baseline value. Statistical significance was set at *P <* 0.05. Data normality was assessed using the Kolmogorov-Smirnov tests for all fatty acid and oxylipin analyses. All statistical assumptions underlying the analyses were evaluated and found to be adequately met. Multiple comparisons were adjusted for using Bonferroni correction to reduce the likelihood of type I errors.

## Results

Table 1 presents the baseline characteristics of the participants included in this study. Unpaired *t*-tests were conducted to assess baseline differences between the 2 groups. The groups were similar overall, except for mean age, HDL-cholesterol, and glucose, which were all significantly higher in the EPA+DHA group. Paired *t*-tests were used to evaluate within-group changes in weight and BMI between preintervention and postintervention, and no statistically significant changes were observed (EPA+DHA: weight *P =* 0.08, BMI *P =* 0.08; CO: weight *P =* 0.42, BMI *P =* 0.46).

**TABLE 1:** Characteristics of study participants at baseline for the EPA+DHA and CO groups.

Variable	Mean ± SD		*P* value^2^
Supplementation group	EPA+DHA (*n* = 10)^1^	CO (*n* = 11)^1^	
Sex M/F (*n*)	1/9	0/11	
Age (y)	49.15 ± 17.45	35.54 ± 13.12	0.06
Weight (kg)	79.69 ± 20.99	73.15 ± 21.32	0.49
BMI (kg/m^2^)	29.81 ± 7.04	27.65 ± 7.38	0.50
Waist (cm)	97.72 ± 21.04	85.07 ± 16.35	0.13
Hip (cm)	107.76 ± 12.08	104.21 ± 17.58	0.60
Body fat (%)	40.39 ± 6.86	35.57 ± 10.81	0.24
Body fat (kg)	34.76 ± 12.12	28.59 ± 15.93	0.33
Lean mass (kg)	49.19 ± 10.79	45.94 ± 6.56	0.41
Cholesterol	5.57 ± 0.98	5.04 ± 1.05	0.25
HDL-cholesterol	1.86 ± 0.42	1.43 ± 0.31	0.02
LDL-cholesterol	3.42 ± 1.09	3.43 ± 0.87	0.98
Glucose	5.95 ± 0.91	4.81 ± 0.34	0.001
Insulin	7.58 ± 2.94	11.69 ± 8.68	0.17
HOMA-IR	1.03 ± 0.41	1.48 ± 1.07	0.23

Abbreviation: CO, corn oil.

1 Data are mean + SD (*n* = 21).

2 *P* values were calculated using an unpaired *t*-test.

### Supplementation with EPA+DHA decreases ω-6 PUFAs across lipid pools

**FIGURE 1:**
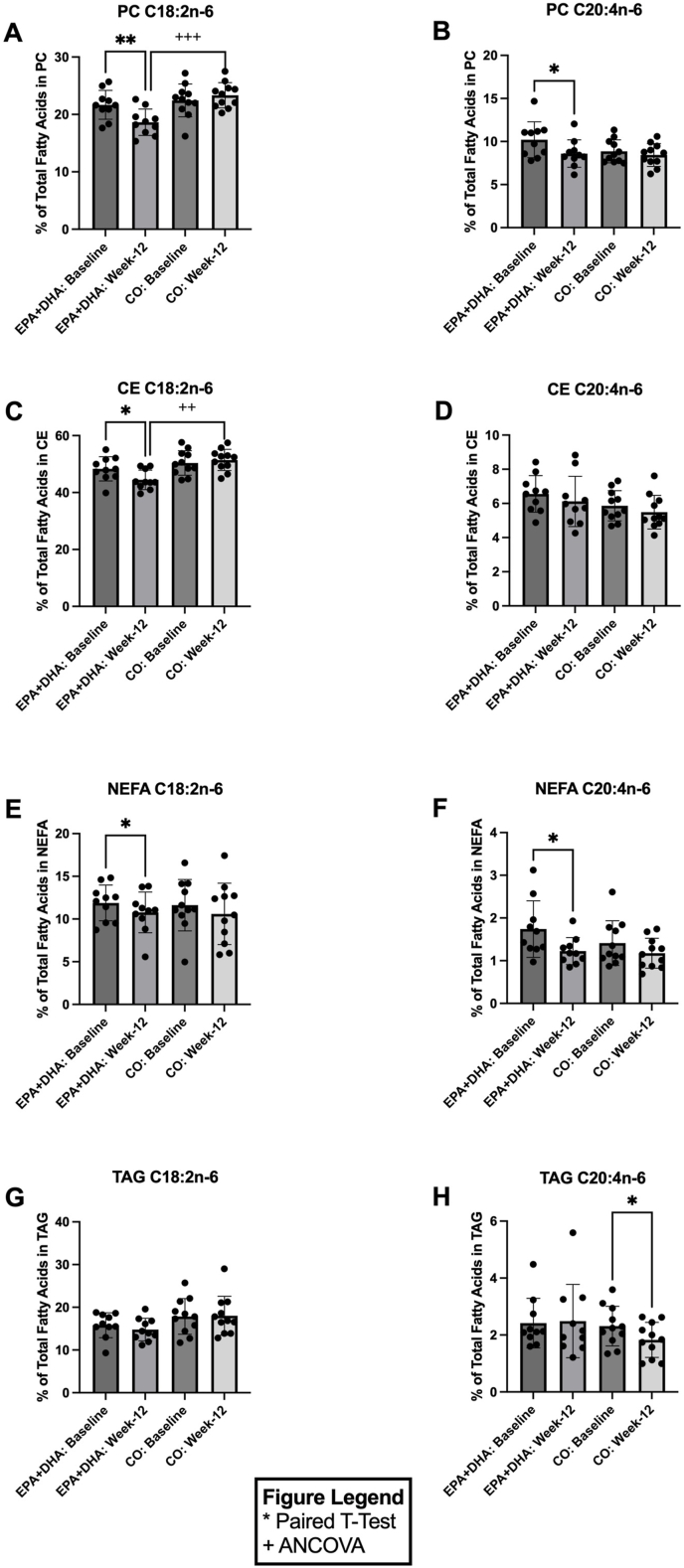
Relative concentrations of omega-6 fatty acids in plasma PC, CE, NEFA, and TAG before (baseline) and after (week 12) EPA+DHA or CO supplementation for *n* = 10 and *n* = 11, respectively. Data are expressed as the percentage of total fatty acids for (A) PC C18:2n–6, (B) PC C20:4n–6, (C) CE C18:2n–6, (D) CE C20:4n–6, (E) NEFA C18:2n–6, (F) NEFA C20:4n–6, (G) TAG C18:2n–6, and (H) TAG C20:4n–6. Error bars represent mean ± SD. Statistical significance was assessed as follows: baseline differences between the 2 groups were evaluated with unpaired *t*-tests; within-group changes from baseline to week 12 were analyzed using paired *t*-tests; and between-group comparisons at week 12 were performed with ANCOVA (∗/^+^*P* < 0.05, ∗∗/^++^*P* < 0.01,∗∗∗/^+++^*P* < 0.001, ∗∗∗∗/^++++^*P* < 0.0001). ANCOVA, Analysis of covariance; CE, cholesteryl esters; CO, corn oil; NEFA, nonesterified fatty acids; PC, phosphatidylcholines; TAG, triglycerides.

Figure 1 shows the percent abundance of key ω-6 fatty acids in the PC, CE, NEFA, and TAG pools (Figure 1A–H). In the PC pool, 12 wk of EPA+DHA supplementation led to significant decreases in ω-6 PUFAs 18:2n-6 (LA) by 0.86-fold [*P =* 0.002; confidence interval (CI): (–4.6, –1.4); Figure 1A] and 20:4n–6 (AA) by 0.84-fold [*P =* 0.012; CI: (–2.8, –0.4); Figure 1B] when compared with baseline. At week 12, PC 18:2n–6 was significantly higher in the CO group [1.25-fold, *P* < 0.001; CI: EPA+DHA (17.9, 20.6) and CO (21.8, 24.5); Figure 1A] compared with the EPA+DHA group at week 12. In the CE pool, in response to 12 wk of EPA+DHA supplementation, 18:2n–6 decreased 0.92-fold [*P =* 0.02; CI: (–6.9, –0.8); Figure 1C] when compared with baseline. At week 12, CE 18:2n–6 was significantly higher in the CO group [1.16-fold, *P =* 0.002; CI: EPA+DHA (43.6, 47.7) and CO (48.7, 53.0); Figure 1C] compared with the EPA+DHA group at week 12. In the NEFA pool, 12 wk of EPA+DHA supplementation decreased ω-6 PUFAs 18:2n–6 by 0.91-fold [*P =* 0.05; CI: (–2.2, –0.01); Figure 1E] and C20:4n–6 by 0.70-fold (*P =* 0.01; CI: (–0.9, –0.1); Figure 1F] when compared with baseline. In the TAG pool, the levels of ω-6 PUFAs remained unchanged after EPA+DHA supplementation when compared with baseline. However, TAG 20:4n–6 decreased 0.79-fold after 12 wk of CO supplementation compared with baseline [*P =* 0.01; CI: (–0.8, –0.1); Figure 1H]. No significant changes were observed in PC, CE, and NEFA pool ω-6 fatty acid compositions in response to 12-wk CO supplementation when compared with baseline (Figure 1A–F).

### Supplementation with EPA+DHA increases ω-3 PUFAs across lipid pools

**FIGURE 2:**
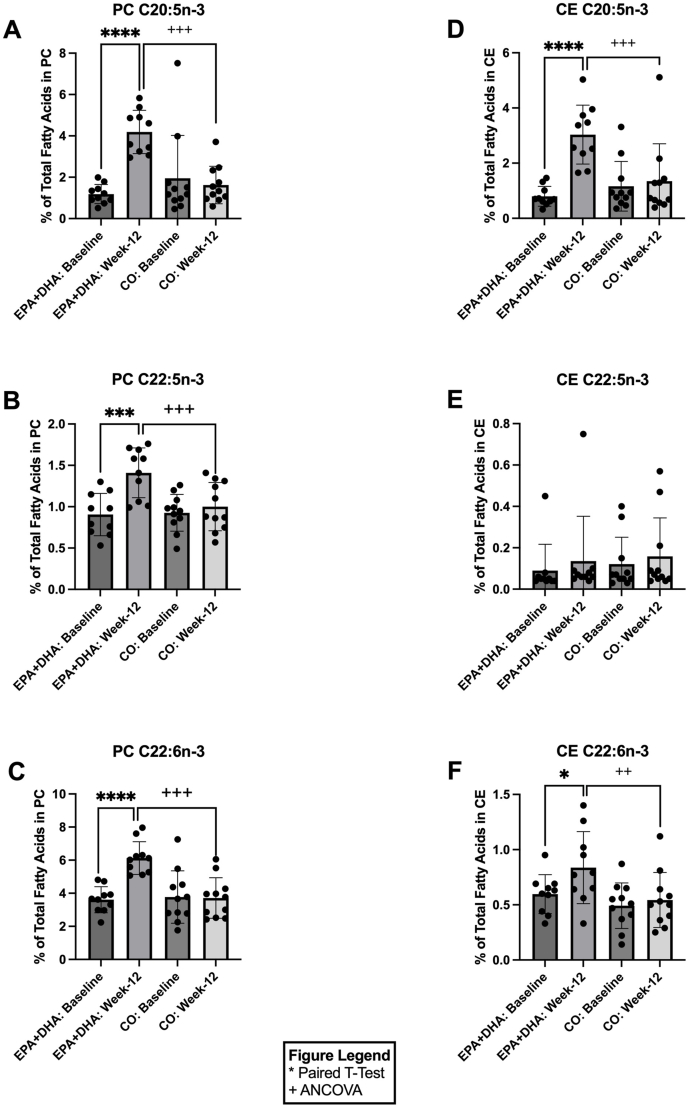
Relative concentrations of omega-3 fatty acids in plasma PC and CE before (baseline) and after (week 12) EPA+DHA or CO supplementation for *n* = 10 and *n* = 11, respectively. Data are expressed as the percentage of total fatty acids for: (A) PC C20:5n–3, (B) PC C22:5n–3, (C) PC C22:6n–3, (D) CE C20:5n–3, (E) CE C22:5n–3, and (F) CE C22:6n–3. Error bars represent mean ± SD. Statistical significance was assessed as follows: baseline differences between the 2 groups were evaluated with unpaired *t*-tests; within-group changes from baseline to week 12 were analyzed using paired *t-*tests; and between-group comparisons at week 12 were performed with ANCOVA (∗/^+^*P* < 0.05, ∗∗/^++^*P* < 0.01,∗∗∗/^+++^*P* < 0.001, ∗∗∗∗/^++++^*P* < 0.0001). ANCOVA, analysis of covariance; CE, cholesteryl esters; CO, corn oil; PC, phosphatidylcholines.

Figure 2 shows the percent abundance of key ω-3 fatty acids in the PC and CE pools (Figure 2A–F). In the PC pool, in response to 12 wk of EPA+DHA supplementation, the long-chain ω-3 PUFAs 20:5n–3 (EPA), 22:5n–3 (DPA), and 22:6n–3 (DHA) increased by 3.53-fold [*P* ≤ 0.0001; CI: (2.2, 3.8); Figure 2A], 1.56-fold [*P =* 0.0007; CI: (0.3, 0.7); Figure 2B], and 1.70-fold [*P* ≤ 0.0001; CI: (1.9, 3.2); Figure 2C], respectively, when compared with baseline. At week 12, the EPA+DHA group had significantly higher levels of 20:5n–3 [2.58-fold, *P* ≤ 0.001; CI: EPA+DHA (3.6, 4.7) and CO (0.8, 2.0); Figure 1A], 22:5n–3 [1.41-fold, *P* ≤ 0.001; EPA+DHA (1.2, 1.6) and CO (0.8, 1.1); Figure 1B], and 22:6n–3 [1.65-fold, *P* ≤ 0.001; CI: EPA+DHA (5.7, 6.6) and CO (3.0, 3.9); Figure 1C] compared with the CO group at week 12. In the CE pool, in response to 12 wk of EPA+DHA supplementation, 20:5n–3 and 22:6n–3 increased 3.81-fold [*P* ≤ 0.0001; CI: (1.6, 2.9); Figure 2D] and 1.40-fold [*P =* 0.03; CI: (0.02, 0.5); Figure 2F], respectively, when compared with baseline. At week 12, significantly higher levels of CE 20:5n–3 [2.24-fold, *P* ≤ 0.001; CI: EPA+DHA (2.7, 3.9) and CO (0.3, 1.6); Figure 2D] and 22:6n–3 [1.55-fold, *P =* 0.01; CI: EPA+DHA (0.06, 0.2) and CO (0.1, 0.2); Figure 2F] were observed in the EPA+DHA group compared with the CO group at week 12. No significant changes were observed in the PC or CE pool ω-3 fatty acid compositions in response to 12 wk of CO supplementation when compared with baseline (Figure 2A–F).

Supplemental Figure 2 presents the SFAs and MUFAs in the PC and CE pools (Supplemental Figure 2A–H). For the PC pool, after 12 wk of EPA+DHA supplementation, 18:1n–9 decreased 0.94-fold (*P =* 0.040, CI: –1.3, –0.06; Supplemental Figure 2D) when compared with baseline. All other PC SFAs and MUFAs remained unchanged. For the CE pool, after 12 wk of EPA+DHA supplementation, 16:0 increased by 1.08-fold [*P =* 0.004, CI: (0.4, 1.6); Supplemental Figure 2F]. Additionally, at week 12 (postsupplementation), the EPA+DHA group had significantly higher levels of CE 16:0 compared with the CO group at week 12 [1.12-fold, *P =* 0.002, CI: EPA+DHA (12.9, 14.2) and CO (11.2, 12.5); Supplemental Figure 2F]. All other SFAs and MUFAs remained unchanged.

**FIGURE 3:**
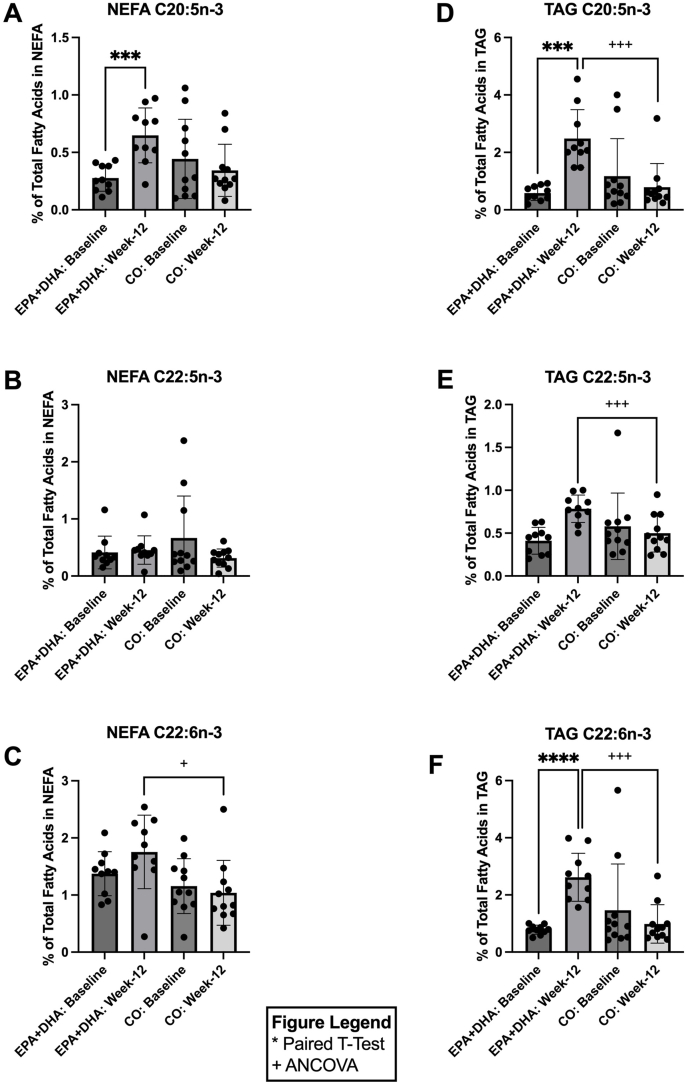
Relative concentrations of omega-3 fatty acids in plasma NEFA and TAG before (baseline) and after (week-12) EPA+DHA or CO supplementation for *n* = 10 and *n* = 11, respectively. Data are expressed as the percentage of total fatty acids for: (A) NEFA C20:5n–3, (B) NEFA C22:5n–3, (C) NEFA C22:6n–3, (D) TAG C20:5n–3, (E) TAG C22:5n–3, and (F) TAG C22:6n–3. Error bars represent mean ± SD. Statistical significance was assessed as follows: baseline differences between the 2 groups were evaluated with unpaired *t*-tests; within-group changes from baseline to week 12 were analyzed using paired *t*-tests; and between-group comparisons at week 12 were performed with ANCOVA (∗/^+^*P* < 0.05, ∗∗/^++^*P* < 0.01,∗∗∗/^+++^*P* < 0.001, ∗∗∗∗/^++++^*P* < 0.0001). ANCOVA, analysis of covariance; CO, corn oil; NEFA, nonesterified fatty acids; TAG, triglycerides.

Figure 3 shows the percent abundance of key ω-3 fatty acids in the NEFA and TAG pools (Figure 3A–F). In the NEFA pool, in response to 12 wk of EPA+DHA supplementation, 20:5n–3 significantly increased 2.34-fold [*P =* 0.0002; CI: (0.2, 0.5); Figure 3A] when compared with baseline. At week 12, NEFA 22:6n–3 [2.66-fold, *P =* 0.02; CI: EPA+DHA (0.4, 0.6) and CO (0.2, 0.4); Figure 3C] levels were significantly higher in the EPA+DHA group in comparison to the CO group at week 12. In the TAG pool, in response to 12 wk of EPA+DHA, 20:5n–3 and 22:6n–3 increased by 4.27-fold [*P =* 0.0004; CI: (1.1, 2.7); Figure 3D] and 3.34-fold [*P* ≤ 0.0001; CI: (1.2, 2.4); Figure 3F], respectively, when compared with baseline. At week 12 (postsupplementation), the EPA+DHA group had significantly higher levels of 20:5n–3 [3.15-fold, *P* ≤ 0.001; CI: EPA+DHA (2.1, 3.0) and CO (0.03, 1.0); Figure 3D], 22:5n–3 [1.57-fold, *P* ≤ 0.001; CI: EPA+DHA (0.7, 0.9) and CO (0.3, 0.6); Figure 3E], and 22:6n–3 [2.66-fold, *P* ≤ 0.001; CI: EPA+DHA (2.2, 3.1) and CO (0.4, 1.2); Figure 3F] compared with the CO group at week 12. No significant changes were observed in the NEFA and TAG pool ω-3 fatty acid compositions in response to 12 wk of CO supplementation when compared with baseline.

Supplemental Figure 3 presents the SFAs and MUFAs in the NEFA and TAG pool (Supplemental Figure 3A–H). All the NEFA SFAs and MUFAs remained unchanged. However, in the TAG pool, after 12 wk of EPA+DHA supplementation, 18:0 increased by 1.20-fold [*P =* 0.03, CI: (0.1, 1.4;) Supplemental Figure 3G] whereas 18:1n–9 decreased by 0.90-fold [*P =* 0.04, CI: (–7.2, –0.02); Supplemental Figure 3H] when compared with baseline. All other SFAs and MUFAs remained unchanged.

### Supplementation with EPA+DHA increases plasma anti-inflammatory oxylipins and decreases proinflammatory oxylipins

**FIGURE 4:**
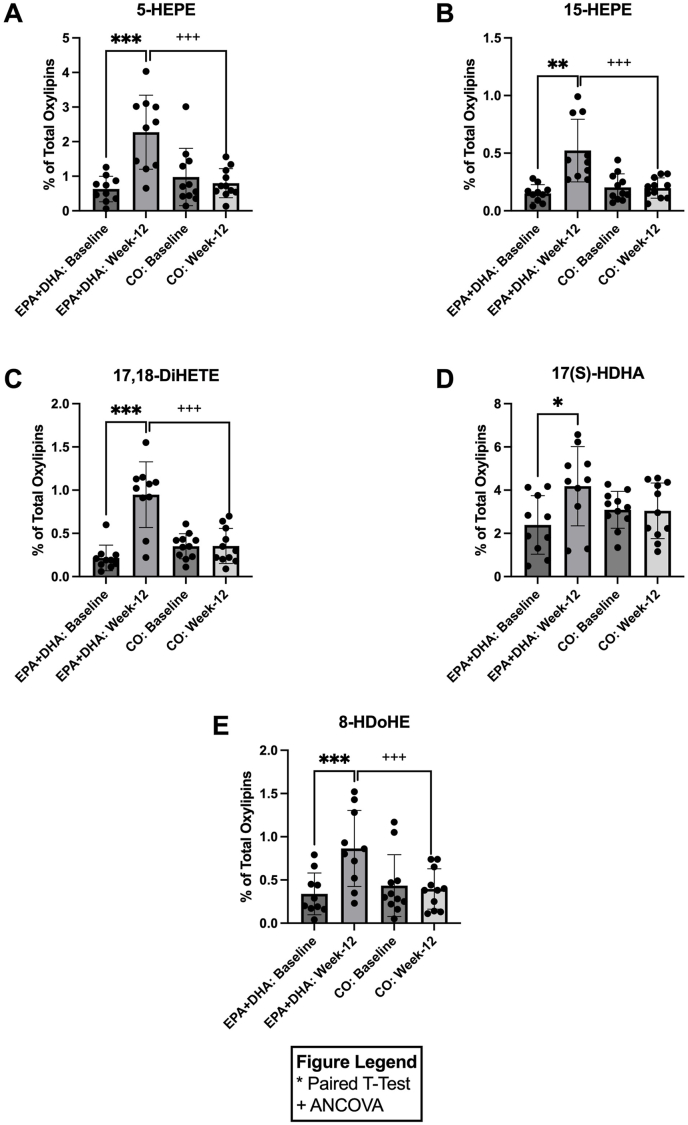
Concentrations of significant omega-3 derived oxylipins before (baseline) and after (week 12) EPA+DHA or CO supplementation for *n* = 10 and *n* = 11, respectively. Data are expressed as the percentage of total oxylipins for: (A) 5-HEPE, (B) 15-HEPE, (C) 17,18-DiHETE, (D) 17(S)-HDHA, and (E) 8-HDoHE. Error bars represent mean ± SD. Statistical significance was assessed as follows: baseline differences between the 2 groups were evaluated with unpaired *t*-tests; within-group changes from baseline to week 12 were analyzed using paired *t*-tests; and between-group comparisons at week 12 were performed with ANCOVA (∗/^+^*P* < 0.05, ∗∗/^++^*P* < 0.01,∗∗∗/^+++^*P* < 0.001, ∗∗∗∗/^++++^*P* < 0.0001). ANCOVA, analysis of covariance; CO, corn oil; DiHETE, dihydroxy-eicosatetraenoic acid; HDHA, hydroxy docosahexaenoic acid; HDoHE, hydroxydocosahexaenoic acid; HEPE, hydroxy-eicosapentaenoic acid.

Figure 4 illustrates the change in circulating plasma oxylipins derived from ω-3 PUFAs (Figure 4A–E). In response to 12-wk supplementation with EPA+DHA, there were significant increases in the EPA-derived oxylipins 5-hydroxy-eicosapentaenoic acid (HEPE), 15-HEPE, and 17,18-dihydroxy-eicosatetraenoic acid (DiHETE) by 3.60-fold [*P =* 0.0005; CI: (0.9, 2.3); Figure 4A], 3.46-fold [*P =* 0.001; CI: (0.2, 0.6); Figure 4B), 4.38-fold [*P =* 0.0002; CI: (0.5, 1.0); Figure 4C], respectively, when compared with baseline. DHA-derived oxylipins also increased in response to 12-wk EPA+DHA supplementation with 17(S)-hydroxy docosahexaenoic acid (HDHA) and 8-hydroxydocosahexaenoic acid (HDoHE), increasing by 1.75-fold [*P =* 0.028; CI: (0.2, 3.5); Figure 4D] and 2.55-fold [*P =* 0.001; CI: (0.3, 0.8); Figure 4E], respectively, when compared with baseline. In response to 12-wk CO supplementation, no significant changes were observed in any of the oxylipins mentioned above (Figure 4A–E). At week 12 (postsupplementation), the EPA+DHA group had significantly higher levels of 5-HEPE [2.85-fold, CI: (0.9, 2.3); *P* ≤ 0.001; Figure 4A], 15-HEPE [2.65-fold, *P <* 0.001; CI: (0.2, 0.6); Figure 4B], 17,18-DiHETE [2.68-fold, *P <* 0.001; CI: (0.5, 1.0); Figure 4C], 8-HDoHE [2.18-fold, *P* ≤ 0.001; CI: (0.3, 0.8); Figure 4E] compared with the CO group at week 12.

**FIGURE 5:**
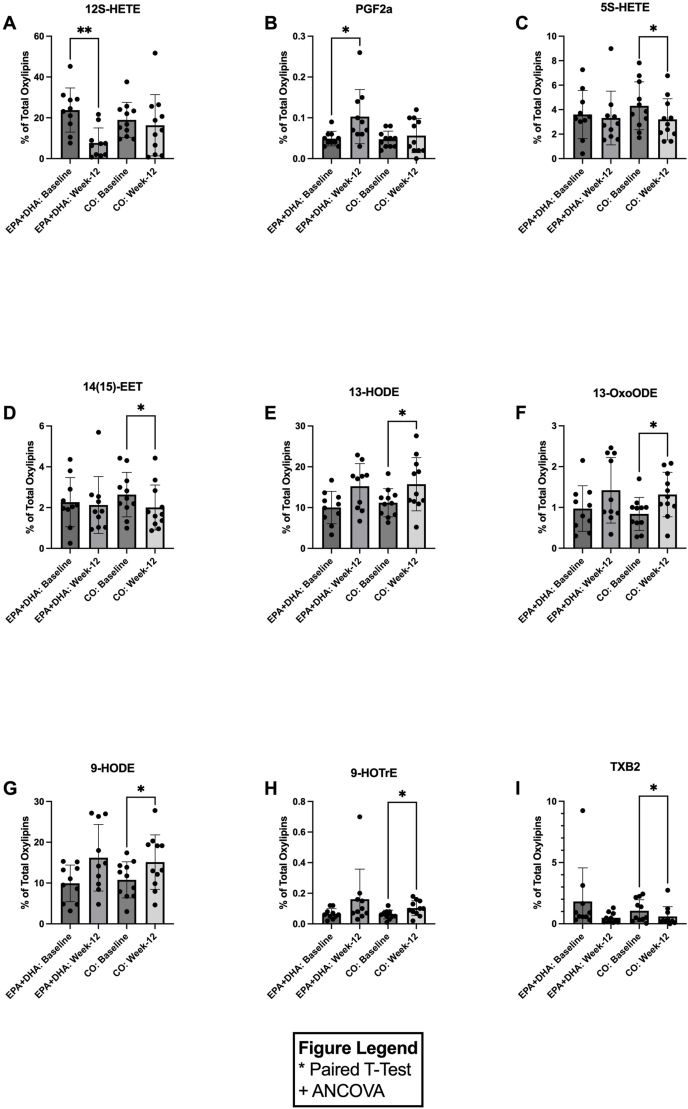
Concentrations of significant omega-6 derived oxylipins before (baseline) and after (week 12) EPA+DHA or corn oil (CO) supplementation for *n* = 10 and *n* = 11, respectively. Data are expressed as the percentage of total oxylipins for: (A) 12-S HETE, (B) PGF2a, (C) 5S-HETE, (D) 14(15)-EET, (E) 13-HODE, (F) 13-OxoODE, (G) 9-HODE, (H) 9-HOTrE, and (I) TXB2. Error bars represent mean ± SD. Statistical significance was assessed as follows: baseline differences between the 2 groups were evaluated with unpaired *t*-tests; within-group changes from baseline to week 12 were analyzed using paired *t*-tests; and between-group comparisons at week 12 were performed with ANCOVA (∗/^+^*P* < 0.05, ∗∗/^++^*P* < 0.01,∗∗∗/^+++^*P* < 0.001, ∗∗∗∗/^++++^*P* < 0.0001). ANCOVA, analysis of covariance; CO, corn oil; EET, epoxyeicosatrienoic acid; HETE, hydroxyeicosatetraenoic acid; HODE, hydroxyoctadecadienoic acid; HOTrE, hydroxyoctadecatrienoic acid; OxoODE, octadecadienoic acid; PGF, prostaglandin; TXB2,thromboxane B2.

Figure 5 illustrates the changes in circulating plasma oxylipins derived from ω-6 PUFAs (Figure 5A–I). In response to 12-wk supplementation with EPA+DHA, there was a significant decrease in the AA-derived oxylipin 12-HETE by 0.32-fold [*P =* 0.0075, CI: (–26.7, –5.5); Figure 5A], and a significant increase in AA-derived oxylipin PGF2 by 2.10-fold [*P =* 0.0275, CI: (0.007, 0.1); Figure 5B] when compared with baseline. In response to 12-wk supplementation with CO, there was a decrease in AA-derived compounds 5S-HETE, 14(15)-epoxyeicosatrienoic acid (EET), and thromboxane B2 (TXB2) by 0.74-fold [*P =* 0.049, CI: (0.007, 0.1); Figure 5C[, 0.76-fold ]*P =* 0.026, CI: (–1.2, –0.09); Figure 5D], and 0.56-fold [*P =* 0.046, CI: (–0.9, –0.01); Figure 5I[, respectively, when compared with baseline. Conversely, in response to 12-wk supplementation with CO, there was a significant increase in AA-derived compounds 13-HODE, 13-octadecadienoic acid (OxoODE), 9-HODE, and 9-hydroxyoctadecatrienoic acid (HOTrE) by 1.41-fold [*P =* 0.026, CI: (0.7, 8.4); Figure 5E], 1.56-fold ]*P =* 0.0332, CI: (0.04, 0.9); Figure 5F], 1.40-fold ]*P =* 0.045, CI: (0.1, 8.6); Figure 5G], and 1.67-fold [*P =* 0.02, CI: (0.01, 0.07); Figure 5H], respectively, when compared with baseline. We also analyzed additional oxylipins, which did not change with EPA+DHA or CO relative to baseline or at week 12, comparing EPA+DHA and CO (Supplemental Figures 4 and 5).

## Discussion

The pattern of EPA and DHA incorporation across various plasma lipid pools highlights the complex nature of ω-3 PUFA handling. After supplementation of 1.1 g EPA + 0.8 g DHA daily for 12 wk, there were significant increases in EPA and DHA in the PC pool relative to baseline. These findings align with an existing study in which middle-aged males received 1.8 g EPA and 0.3 g DHA for 8 wk [19]. In that study, the proportion of EPA in plasma PC increased by a 3.57-fold-change, closely matching our observed fold-change of 3.53-fold for EPA relative to baseline [19]. Interestingly, while that study reported no significant changes in DHA, our study showed a 1.70-fold increase from baseline [19]. These differences are likely due to the lower amount of DHA in the supplement compared with the current study (0.3 g compared with 0.8 g) and possibly the difference in intervention duration between the 2 studies (8 wk compared with 12 wk) [19].

After EPA+DHA supplementation, the relative abundance of EPA and DHA in the CE pool increased relative to baseline. The previously mentioned study among middle-aged males reported a rise in CE-associated EPA from 1.2% to 4.5% of total fatty acids**,** supporting the efficacy of supplementation in enriching EPA across lipid pools [19]. EPA supplementation decreases cholesteryl ester transfer protein (CETP) activity, which raises HDL-cholesterol while lowering LDL-cholesterol and VLDL-cholesterol, thereby supporting cardiovascular health [20]. Our finding that DHA also increased in this pool contrasts with earlier reports that described selective incorporation of EPA over DHA in CEs [21,22]. This difference in enrichment may reflect variations in enzymatic specificity governing cholesterol esterification and distinct metabolic turnover rates of EPA and DHA within lipid pools [15].

Although literature assessing the incorporation of fatty acids within TAG is sparse, on supplementation, ω-3s displace both MUFAs and SFAs within this pool [23]. A supplementation study found that when plasma concentrations of both EPA and DHA increase, there are marked steric changes in the TAG molecule, resulting in preferential positioning of these ω-3 fatty acids at the sn-1 and sn-3 positions [24]. These findings align with our research, where EPA+DHA showed significant increases in the TAG pool compared with their baseline levels after 12 wk of EPA+DHA supplementation.

The NEFA pool showed an increase in EPA, with no reported increases in DHA relative to baseline after EPA+DHA supplementation. Interestingly, when comparing postsupplementation (week 12) values between groups, we observed significantly lower DHA levels in the NEFA pool in the CO group compared with the EPA+DHA group. Because plasma NEFA represents fatty acids released from adipose tissue in the fasting state, these observations suggest limited adipose tissue incorporation of DHA during this 12-wk timeframe compared with EPA [25]. Previous work demonstrated that DHA supplementation for 6 wk leads to a marked rise in DHA within the NEFA pool, with a 212% increase after low-dose supplementation (0.75 g/d) [25]. The aforementioned research exclusively examined healthy Asian Indians, whereas our study focused on white females, which could influence metabolism due to genetic differences [25]. There are known genetic variants in the cluster determining long-chain PUFA levels across tissues [26]. Around 80% of African Americans have 2 copies of the alleles linked to elevated AA and reduced EPA/DHA, compared with around 45% of European Americans [26].

A central mechanism by which long-chain ω-3 PUFAs lower inflammation is through the decrease in ω-6 PUFAs, especially AA [6]. In the PC and NEFA pool, significant decreases in both LA and AA were observed for the EPA+DHA group relative to baseline. This aligns with existing studies, which have shown that plasma PC exhibits a dose-dependent decrease in oleic acid and 3 n-6 PUFAs (LA, di-homo-γ-LA, and AA) as EPA and DHA dosage and incorporation increase [23]. Plasma PC fatty acid composition is believed to be closely related to that of cell and organelle membranes [27]. Our findings align with recent studies showing that ω-3 PUFA supplementation (2 g EPA and 1 g DHA daily for 12 wk) can significantly increase EPA and DHA in mitochondrial membranes while directly displacing ω-6 PUFAs across several phospholipid populations, suggesting a competitive relationship for membrane incorporation [27]. We previously found a modest decrease in serum AA with a marine oil supplement in adults with obesity, findings consistent with those of the current study [28].

After supplementation, we observed significantly lower LA levels within the CE pool in the EPA+DHA group compared with the CO group at week 12. This finding is particularly relevant, as previous research has shown that CEs enriched with ω-6 PUFAs are more susceptible to oxidation, potentially contributing to the progression of atherosclerosis [29]. The reduced LA content in the CE pool after ω-3 PUFA supplementation may therefore represent a potential cardioprotective mechanism, as lower levels of oxidized LDL have been associated with a reduced risk of cardiovascular disease [29]. We also noticed changes in select SFAs and MUFAs after 12 wk of EPA+DHA supplementation. This is an interesting finding that suggests EPA+DHA may indirectly alter the metabolism and remodeling of other fatty acid classes, warranting further study.

After supplementation with EPA+DHA, EPA-derived oxylipins, 5-HEPE, 15-HEPE, and 17,18-DiHETE, were substantially increased relative to baseline. 5-HEPE plays a role in endocrine functions and can induce regulatory T-cells in murine models [29,30]. 15-HEPE is known to decrease neutrophil migration during inflammation [29]. Although the role of 17,18-DiHETE is not well understood, higher levels are found in individuals after ω-3 PUFA supplementation compared with baseline [31]. DHA-derived oxylipins, 17(S)-HDHA and 8-HDoHE, increased on EPA+DHA intervention relative to baseline. 17(S)-HDHA has implications in pain modulation, with high circulating levels correlating with increased heat pain thresholds and reduced pain scores in patients with osteoarthritis [32].

We also observed a significant increase in the proinflammatory oxylipin PGF2α with EPA+DHA relative to baseline [33]. This finding is particularly intriguing, as PGF2α is derived from AA, which typically competes with EPA and DHA for the same enzymatic pathways [33]. In our previous work, similar increases in PGF2α after marine oil supplementation were reported, which is likely driven by small amounts of AA in the oil [28]. Additionally, after EPA+DHA supplementation, we observed a decrease in the proinflammatory oxylipin, 12S-HETE, relative to baseline. Thus, the lower concentrations of this oxylipin might relate to the lower AA seen after EPA+DHA supplementation in the PC pool. These decreases might be functionally significant. 12S-HETE plays a role in cell proliferation and metastasis [34].

We observed that 12-wk supplementation with CO resulted in significant changes in several oxylipins. 5S-HETE, 14(15)-EET, and TXB2 all decreased following supplementation. A prior study found that higher intake of LA resulted in a decrease in PC AA in HDL, indicating reduced AA availability as a substrate for eicosanoid synthesis [35]. This is consistent with previous work in Atlantic salmon, where increasing dietary LA led to decreased TXB2 and other oxylipins in stimulated gill cells [33]. We also observed increases in oxidized LA metabolites, including 13-HODE, 13-OxoODE, 9-HODE, and 9-HOTrE, following supplementation with corn oil. These oxidized LA metabolites are formed from LA through the action of oxidoreductases such as glutathione peroxidase and hydroxy fatty acid dehydrogenase [36]. Given that our research showed CO supplementation to result in higher LA content in both PC and CE, this provides a direct explanation for the increased concentrations of these oxidized metabolites in circulation.

Our findings generally support the notion that EPA and DHA work through a dual mechanism, enhancing the synthesis of anti-inflammatory oxylipins while concurrently inhibiting the synthesis of proinflammatory oxylipins, resulting in a shift toward an overall anti-inflammatory profile. Notably, we did not find an increase in EPA and DHA-derived oxylipins of the specialized proresolving mediator (SPM) family. This may be due to the limited production and/or stability of SPMs. Previously, we observed an increase in some, but not most, SPMs in scWAT with a marine oil supplement in adults with obesity, consistent with several other studies [37]. For example, patients with coronary artery disease have decreased SPMs at baseline compared with healthy individuals [38]. Notably, on EPA+DHA treatment for 1 y, only certain SPMs that enhanced macrophage-based clot phagocytosis increased, and there was no overall increase in ω-3-derived SPM family oxylipins [38].

Compliance to the intervention was high at 90% in those receiving EPA+DHA and 89% in those receiving corn oil. However, this study has several limitations, as the analyses were exploratory. This study is limited by its small sample size, with only 21 participants. This modest number of participants reduces statistical power and increases the likelihood of type II error [39]. However, multiple comparisons were adjusted for using Bonferroni correction to reduce the likelihood of type I errors. In addition, the small sample size limits generalizability to broader populations [39]. These findings should be validated in larger, more adequately powered studies designed to test these outcomes.

Individuals of healthy weight and those living with obesity were included in the study, which is reflected in the mean cohort BMI of 28.71 kg/m^2^. This may suggest the findings are most relevant to people living with overweight; however, as both individuals living at a healthy weight and those living with obesity were included, the findings are relevant to both these populations. Additionally, it is important to note that the baseline cholesterol and glucose levels differed significantly between the 2 groups, likely reflecting the inclusion of both individuals living at a healthy weight and those living with obesity. Because this was an exploratory analysis using participants with complete plasma fatty acid and oxylipin data, we did not prescreen or stratify the groups by metabolic variables. Differences in these 2 parameters and BMI status could have introduced metabolic confounding. In addition, the preexisting gut microbiome among our study participants served as an uncontrolled variable that may have influenced the observed responses [40]. Supplementation with long-chain ω-3 PUFAs has been shown to increase beneficial intestinal bacteria, including *Lactobacillus* and *Bifidobacterium*, whereas decreasing the proinflammatory species associated with the Western diet [41]. These changes enhance gut barrier integrity, reduce systemic inflammation, and improve lipid metabolism, contributing to the observed health benefits [41]. Variation of microbiome profiles at baseline may influence individual response to supplementation [40,41]. Studies have shown that gut microorganisms such as *Bifidobacterium* influence fatty acid metabolism and epithelial uptake into the intestine [41].

We did not account for the genetic variation that affects ω-3 PUFA metabolism [40]. In addition to the genetic variations mentioned earlier, a prior research study found that specific fatty acid desaturase (FADS) gene variants in Hispanic populations reduce unsaturated fatty acid synthesis, and polymorphisms in the CYP 450 enzymes and the apolipoprotein E (APOE) genotype influence EPA and DHA metabolism and oxylipin production [42]. Sex differences also impact PUFA metabolism, another limitation of this study [40]. Although we only had 1 male participant, future studies should investigate sex differences, as research has shown that females accumulate ω-3 PUFA-derived oxylipins in plasma compared with males, despite no evident differences in precursor PUFA accumulation between sexes [43]. Additionally, concentrations of 16 of 62 measured oxylipins, including 5 of 12 DHA-derived oxylipins, were higher in females than in males, suggesting sex differences in oxylipin metabolism [43]. Thus, there is a need for studies that consider genotype and sex as variables.

In summary, as chronic low-grade inflammatory conditions continue to rise globally, our findings offer insights into the molecular pathways through which ω-3 PUFAs may exert their beneficial effects, highlighting the potential of nutritional interventions as therapeutic agents. We highlight limitations, including the study design, sample size, and potential influences from gut microbiome variability, genetic, and sex differences in ω-3 PUFA metabolism, which require consideration for translating these findings to broader populations.

## Author contributions

The authors’ responsibilities were as follows – NB: contributed to data curation, data analysis, writing – original draft, writing – review and editing; SRS: contributed to conceptualization, formal analysis, data curation, supervision, writing – review and editing; CEC: contributed to project administration, formal analysis, and writing – review and editing; EAM: contributed to conceptualization and writing – review and editing; PSN: contributed to project administration, formal analysis, and writing – review and editing; CP-C: contributed to project administration, investigation, and writing – review and editing; PCC: contributed to conceptualization, funding acquisition, supervision, and writing – review and editing; HLF: contributed to conceptualization, investigation, formal analysis, data curation, supervision, writing – review and editing; and all authors: contributed to the article and approved the submitted version.

## Funding

This study was supported by National Institutes of Health (NIH) P30DK056350 and R01ES031378 (SRS), European Commission, Seventh Framework Programme Grant Number 244995 (PCC), National Institute for Health and Care Research (NIHR) Southampton Biomedical Research Centre Grant Number NIHR203319 (HLF).

## Conflict of interest

HLF reports financial support was provided by NIHR Southampton Biomedical Research Centre. PCC reports financial support was provided by European Commission Seventh Framework Programme for Research and Technological Development. SRS reports financial support was provided by NIH. SRS is an editorial board member for *Journal of Nutrition*. PCC is Associate Editor for the *Journal of Nutrition*. Given their roles as editorial members, neither SRS or PCC no involvement in the peer review of this article and had no access to information regarding its peer review. Full responsibility for the editorial process for this article was delegated to another journal editor. If there are other authors, they declare that they have no known competing financial interests or personal relationships that could have appeared to influence the work reported in this paper.

## References

[1] Placha D., Jampilek J. (2021). Chronic inflammatory diseases, anti-inflammatory agents and their delivery nanosystems. Pharmaceutics.

[2] Buttorff C., Ruder T., Bauman M. (2017). https://www.rand.org/pubs/tools/TL221.html.

[3] Cordain L., Eaton S.B., Sebastian A., Mann N., Lindeberg S., Watkins B.A. (2005). Origins and evolution of the Western diet: health implications for the 21st century. Am. J. Clin. Nutr..

[4] Simopoulos A.P. (2002). The importance of the ratio of omega-6/omega-3 essential fatty acids. Biomed. Pharmacother..

[5] Borja-Magno A., Guevara-Cruz M., Flores-López A., Carrillo-Domínguez S., Granados J., Arias C. (2023). Differential effects of high dose omega-3 fatty acids on metabolism and inflammation in patients with obesity: eicosapentaenoic and docosahexaenoic acid supplementation. Front. Nutr..

[6] Djuricic I., Calder P.C. (2021). Beneficial outcomes of omega-6 and omega-3 polyunsaturated fatty acids on human health: an update for 2021. Nutrients.

[7] Shaikh S.R., Beck M.A., Alwarawrah Y., MacIver N.J. (2024). Emerging mechanisms of obesity-associated immune dysfunction. Nat. Rev. Endocrinol..

[8] Kavyani Z., Musazadeh V., Fathi S., Hossein Faghfouri A., Dehghan P., Sarmadi B. (2022). Efficacy of the omega-3 fatty acids supplementation on inflammatory biomarkers: an umbrella meta-analysis. Int. Immunopharmacol..

[9] Fisk H.L., Childs C.E., Miles E.A., Ayres R., Noakes P.S., Paras-Chavez C. (2022). Modification of subcutaneous white adipose tissue inflammation by omega-3 fatty acids is limited in human obesity-a double blind, randomised clinical trial. EBioMedicine.

[10] Knuplez E., Sturm E.M., Marsche G. (2021). Emerging role of phospholipase-derived cleavage products in regulating eosinophil activity: focus on lysophospholipids, polyunsaturated fatty acids and eicosanoids. Int. J. Mol. Sci..

[11] Choi S.H., Sviridov D., Miller Y.I. (2017). Oxidized cholesteryl esters and inflammation. Biochim. Biophys. Acta Mol. Cell Biol. Lipids..

[12] Deng Q., Du L., Zhang Y., Liu G. (2021). NEFAs influence the inflammatory and insulin signaling pathways through TLR4 in primary calf hepatocytes in vitro. Front. Vet. Sci..

[13] Zeng C.M., He J., Wang D.C., Xie H. (2025). Association between triglyceride levels and rheumatoid arthritis prevalence in women: a cross-sectional study of NHANES (1999–2018). BMC Womens Health.

[14] Simopoulos A.P. (2016). An increase in the omega-6/omega-3 fatty acid ratio increases the risk for obesity. Nutrients.

[15] Surette M.E. (2008). The science behind dietary omega-3 fatty acids. CMAJ.

[16] Fisk H.L., Childs C.E., Miles E.A., Ayres R., Noakes P.S., Paras-Chavez C. (2021). Dysregulation of endocannabinoid concentrations in human subcutaneous adipose tissue in obesity and modulation by omega-3 polyunsaturated fatty acids. Clin. Sci. (Lond.)..

[17] Fisk H.L., West A.L., Childs C.E., Burdge G.C., Calder P.C. (2014). The use of gas chromatography to analyze compositional changes of fatty acids in rat liver tissue during pregnancy. J. Vis. Exp..

[18] Armstrong M., Manke J., Nkrumah-Elie Y., Shaikh S.R., Reisdorph N. (2020). Improved quantification of lipid mediators in plasma and tissues by liquid chromatography tandem mass spectrometry demonstrates mouse strain specific differences. Prostaglandins Other Lipid Mediat.

[19] Yusof H.M., Miles E.A., Calder P. (2008). Influence of very long-chain n-3 fatty acids on plasma markers of inflammation in middle-aged men, Prostaglandins Leukot. Essent. Fatty Acids.

[20] So J., Asztalos B.F., Horvath K., Lamon-Fava S. (2022). Ethyl EPA and ethyl DHA cause similar and differential changes in plasma lipid concentrations and lipid metabolism in subjects with low-grade chronic inflammation. J. Clin. Lipidol..

[21] Oscarsson J., Hurt-Camejo E. (2017). Omega-3 fatty acids eicosapentaenoic acid and docosahexaenoic acid and their mechanisms of action on apolipoprotein B-containing lipoproteins in humans: a review. Lipids Health Dis.

[22] Browning L.M., Walker C.G., Mander A.P., West A.L., Madden J., Gambell J.M. (2012). Incorporation of eicosapentaenoic and docosahexaenoic acids into lipid pools when given as supplements providing doses equivalent to typical intakes of oily fish. Am. J. Clin. Nutr..

[23] Walker C.G., West A.L., Browning L.M., Madden J., Gambell J.M., Jebb S.A. (2015). The pattern of fatty acids displaced by EPA and DHA following 12 months supplementation varies between blood cell and plasma fractions. Nutrients.

[24] Alijani S., Hahn A., Harris W.S., Schuchardt J.P. (2025). Bioavailability of EPA and DHA in humans - a comprehensive review. Prog. Lipid Res..

[25] Conquer J.A., Holub B.J. (1998). Effect of supplementation with different doses of DHA on the levels of circulating DHA as non-esterified fatty acid in subjects of Asian Indian background. J Lipid Res.

[26] Mathias R.A., Pani V., Chilton F.H. (2014). Genetic variants in the FADS gene: implications for dietary recommendations for fatty acid intake. Curr. Nutr. Rep..

[27] Herbst E.A., Paglialunga S., Gerling C., Whitfield J., Mukai K., Chabowski A. (2014). Omega-3 supplementation alters mitochondrial membrane composition and respiration kinetics in human skeletal muscle. J. Physiol..

[28] Al-Shaer A.E., Regan J., Buddenbaum N., Tharwani S., Drawdy C., Behee M. (2022). Enriched marine oil supplement increases specific plasma specialized pro-resolving mediators in adults with obesity. J. Nutr..

[29] Schaller M.S., Zahner G.J., Gasper W.J., Harris W.S., Conte M.S., Hills N.K. (2017). Relationship between the omega-3 index and specialized pro-resolving lipid mediators in patients with peripheral arterial disease taking fish oil supplements. J. Clin. Lipidol..

[30] Onodera T., Fukuhara A., Shin J., Hayakawa T., Otsuki M., Shimomura I. (2017). Eicosapentaenoic acid and 5-HEPE enhance macrophage-mediated Treg induction in mice. Sci. Rep..

[31] Saleh R.N.M., West A.L., Ostermann A.I., Schebb N.H., Calder P.C., Minihane A.M. (2021). APOE genotype modifies the plasma oxylipin response to omega-3 polyunsaturated fatty acid supplementation in healthy individuals. Front. Nutr..

[32] Valdes A.M., Ravipati S., Menni C., Abhishek A., Metrustry S., Harris J. (2017). Association of the resolvin precursor 17-HDHA, but not D- or E- series resolvins, with heat pain sensitivity and osteoarthritis pain in humans. Sci. Rep..

[33] Xu C., You X., Liu W., Sun Q., Ding X., Huang Y. (2015). Prostaglandin F2α regulates the expression of uterine activation proteins via multiple signalling pathways. Reproduction.

[34] Powell W.S., Rokach J. (2015). Biosynthesis, biological effects, and receptors of hydroxyeicosatetraenoic acids (HETEs) and oxoeicosatetraenoic acids (oxo-ETEs) derived from arachidonic acid. Biochim. Biophys. Acta..

[35] Adam O., Tesche A., Wolfram G. (2008). Impact of linoleic acid intake on arachidonic acid formation and eicosanoid biosynthesis in humans, Prostaglandins Leukot. Essent. Fatty Acids.

[36] Ramsden C.E., Ringel A., Feldstein A.E., Taha A.Y., MacIntosh B.A., Hibbeln J.R. (2012). Lowering dietary linoleic acid reduces bioactive oxidized linoleic acid metabolites in humans. Prostaglandins Leukot. Essent. Fatty Acids.

[37] Al-Shaer A.E., Buddenbaum N., Shaikh S.R. (2021). Polyunsaturated fatty acids, specialized pro-resolving mediators, and targeting inflammation resolution in the age of precision nutrition. Biochim. Biophys. Acta Mol. Cell Biol. Lipids..

[38] Gracia Aznar A., Moreno Egea F., Gracia Banzo R., Gutierrez R., Rizo J.M., Rodriguez-Ledo P. (2024). Pro-resolving inflammatory effects of a marine oil enriched in specialized pro-resolving mediators (SPMs) supplement and its implication in patients with Post-COVID Syndrome (PCS). Biomedicines.

[39] Faber J., Fonseca L.M. (2014). How sample size influences research outcomes. Dent. Press J. Orthod..

[40] Shaikh S.R., Bazinet R.P. (2023). Heterogeneity in the response to n-3 polyunsaturated fatty acids. Curr. Opin. Clin. Nutr. Metab. Care..

[41] Fu Y., Wang Y., Gao H., Li D., Jiang R., Ge L. (2021). Associations among dietary omega-3 polyunsaturated fatty acids, the gut microbiota, and intestinal immunity. Mediators Inflamm.

[42] Yang C., Hallmark B., Chai J.C., O'Connor T.D., Reynolds L.M., Wood A.C. (2021). Impact of Amerind ancestry and FADS genetic variation on omega-3 deficiency and cardiometabolic traits in Hispanic populations, Commun. Biol..

[43] Calder P.C. (2021). Sex differences in the plasma accumulation of oxylipins in response to supplemental n-3 fatty acids. J. Nutr..

