# Supplementary material for: Effects of EPA+DHA and Corn Oil Supplementation on PUFA Concentrations across Plasma Lipid Pools and on Downstream Oxylipins: Exploratory Results from a Randomized Controlled Trial in Healthy Humans
Source: J Nutr. 2025 Dec 27;156(2):101274. doi: 10.1016/j.tjnut.2025.101274 (PMC12975358; doi:10.1016/j.tjnut.2025.101274)
Supplement: Multimedia component 1 [file mmc1.docx]

**Supplemental Figures
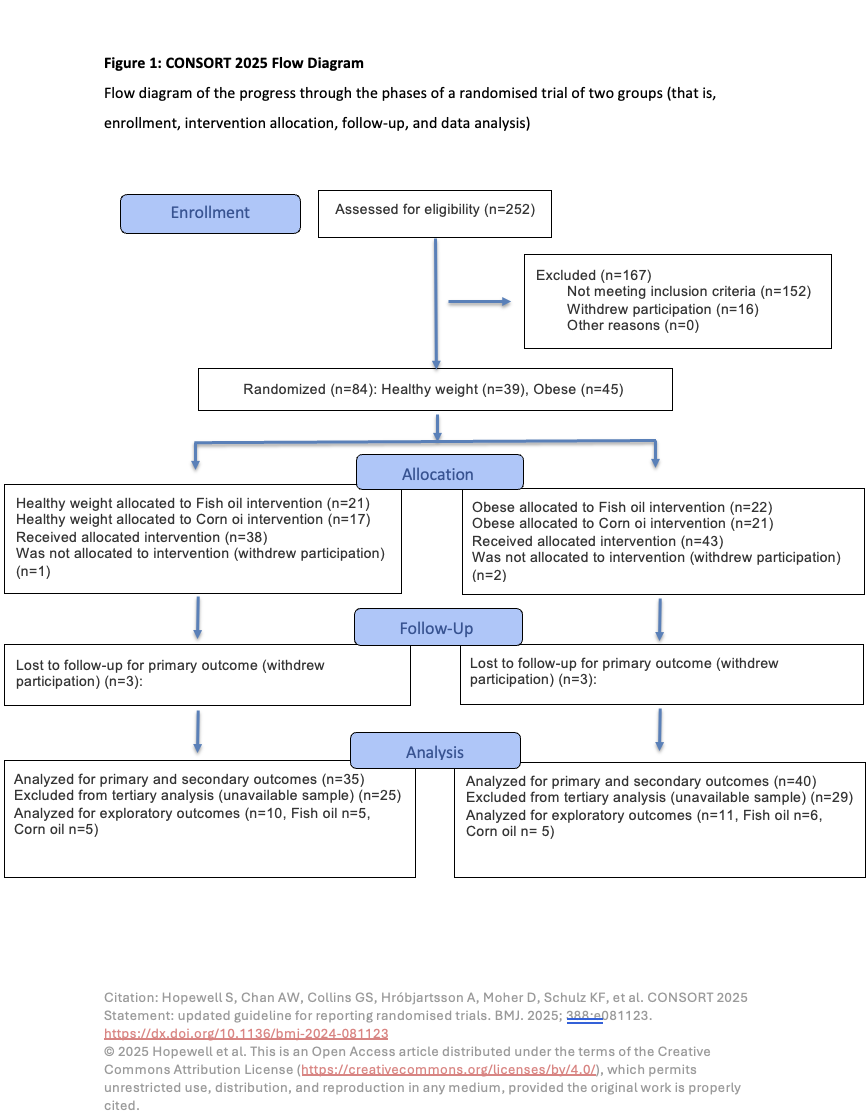
**

Supplemental Figure 1

Supplemental Figure 2

Supplemental Figure 3

Supplemental Figure 4

Supplemental Figure 5

**Supplemental Figure Legends**

**Supplemental Figure 1. CONSORT diagram** depicting the entire study design including sample origin from previous clinical trial.

**Supplemental Figure 2.** Relative concentrations of fatty acids in plasma phosphatidylcholines and cholesterol esters before (baseline) and after (week-12) EPA+DHA or CO supplementation for n=10 and n=11, respectively. Data are expressed as the percentage of total fatty acids for (A) PC C14:0, (B) PC C16:0, (C) PC C18:0, (D) PC C18:1n-9, (E) CE C14:0, (F) CE C16:0, (G) CE C18:0, (H) CE 18:1n-9. Error bars represent mean ± standard deviation. Statistical significance was assessed as follows: baseline differences between the two groups were evaluated with unpaired t-tests; within-group changes from baseline to week 12 were analyzed using paired t-tests; and between-group comparisons at week 12 were performed with ANCOVA (*/^+^p < 0.05, **/^++^p<0.01,***/^+++^p < 0.001, ****/^++++^p < 0.0001). CE, cholesteryl esters; CO, Corn oil; DHA, Docosahexaenoic acid; EPA, Eicosapentaenoic acid; PC, Phosphatidylcholines .

**Supplemental Figure 3.** Relative concentrations of fatty acids in plasma non-esterified fatty acids and triglycerides before (baseline) and after (week-12) EPA+DHA or CO supplementation for n=10 and n=11, respectively. Data are expressed as the percentage of total fatty acids for (A) NEFA C14:0, (B) NEFA C16:0, (C) NEFA C18:0, (D) NEFA C18:1n-9, (E) TAG C14:0, (F) TAG C16:0, (G) TAG C18:0, (H) TAG 18:1n-9. Error bars represent mean ± standard deviation. Statistical significance was assessed as follows: baseline differences between the two groups were evaluated with unpaired t-tests; within-group changes from baseline to week 12 were analyzed using paired t-tests; and between-group comparisons at week 12 were performed with ANCOVA (*/^+^p < 0.05, **/^++^p<0.01,***/^+++^p < 0.001, ****/^++++^p < 0.0001). CO, Corn oil; DHA, Docosahexaenoic acid; EPA, Eicosapentaenoic acid; NEFA, Non-esterified fatty acids; TAG, Triglycerides.

**Supplemental Figure 4.** Relative concentrations of circulating omega-3 derived oxylipins before and after EPA+DHA or CO supplementation for n=10 and n=11, respectively. Data are expressed as the percentage of total oxylipins for (A) 11-HDoHE, (B) 14(S)-HDHA, (C) 19,20-DiHDPA, (D) 9-HOTrE. Error bars represent mean ± standard deviation. Statistical significance was assessed as follows: baseline differences between the two groups were evaluated with unpaired t-tests; within-group changes from baseline to week 12 were analyzed using paired t-tests; and between-group comparisons at week 12 were performed with ANCOVA (*/^+^p < 0.05, **/^++^p<0.01,***/^+++^p < 0.001, ****/^++++^p < 0.0001). CO, Corn oil; DHA, Docosahexaenoic acid; EPA, Eicosapentaenoic acid.

**Supplemental Figure 5.** Relative concentrations of circulating omega-6 derived oxylipins before and after EPA+DHA or CO supplementation for n=10 and n=11, respectively. Data are expressed as the percentage of total oxylipins for (A) 11(12)-EET, (B) 12,13-DiHOME, (C) 12(13)-EpOME, (D) 12-HHTrE, (E) 14,15-DiHETrE, (F) 15S-HETE, (G) 15-HETrE, (H) 20-hydroxy-LTB4, (I) 5,6-DiHETrE, (J) 5-KETE, (K) 8S-HETE, (L) 8-iso-PGF2a, (M) 9(10)-EpOME, (N) 9-HETE, (O) 9-OxoODE, (P) LTB4, (Q) LXA4, (R) PGD2, (S) PGE2, (T) PGJ2. Error bars represent mean ± standard deviation. Statistical significance was assessed as follows: baseline differences between the two groups were evaluated with unpaired t-tests; within-group changes from baseline to week 12 were analyzed using paired t-tests; and between-group comparisons at week 12 were performed with ANCOVA (*/^+^p < 0.05, **/^++^p<0.01,***/^+++^p < 0.001, ****/^++++^p < 0.0001). CO, Corn oil; DHA, Docosahexaenoic acid; EPA, Eicosapentaenoic acid.
